# Maternal Serum Polychlorinated Biphenyl Concentrations across Critical Windows of Human Development

**DOI:** 10.1289/ehp.10086

**Published:** 2007-07-09

**Authors:** Michael S. Bloom, Germaine M. Buck Louis, Enrique F. Schisterman, Aiyi Liu, Paul J. Kostyniak

**Affiliations:** 1 Epidemiology Branch and; 2 Biometry and Mathematical Statistics Branch, Division of Epidemiology, Statistics and Prevention Research, National Institute of Child Health and Human Development, National Institutes of Health, Department of Health and Human Services, Bethesda, Maryland, USA; 3 Department of Biotechnical and Clinical Laboratory Sciences, School of Medicine and Biomedical Sciences, University at Buffalo, the State University of New York, Buffalo, New York, USA

**Keywords:** critical windows, infertility, periconception, persistent organic pollutants, polychlorinated biphenyls (PCBs), pregnancy loss

## Abstract

**Background:**

Few data are available on polychlorinated biphenyl (PCB) concentrations over critical windows of human reproduction and development inclusive of the periconception window.

**Objectives:**

Our goal was to measure changes in PCB concentrations from preconception to pregnancy, through pregnancy, or after a year without becoming pregnant.

**Methods:**

Seventy-nine women planning pregnancies were prospectively enrolled and followed for up to 12 menstrual cycles of attempting pregnancy. Blood specimens were obtained from participating women preconceptionally (*n* = 79), after a positive pregnancy test leading to a live birth (*n* = 54) or pregnancy loss (*n* = 10), at approximately 6 weeks postpartum (*n* = 53), and after 12 unsuccessful cycles (*n* = 9) for toxicologic analysis of 76 PCB congeners. We estimated overall and daily rate of change in PCB concentration (nanograms per gram serum) adjusting for relevant covariates, serum lipids, and baseline PCB concentration.

**Results:**

Significant (*p* < 0.0001) decreases in the mean overall and daily rate of change in PCB concentrations were observed between the preconception and first pregnancy samples for total (–1.012 and –0.034, respectively), estrogenic (–0.444 and –0.016, respectively), and antiestrogenic (–0.106 and –0.004, respectively) PCBs among women with live births. Similar significant decreases in total (–1.452 and –0.085), estrogenic (–0.647 and –0.040), and antiestrogenic (–0.093 and –0.004) PCB concentrations were seen for women with pregnancy losses. No significant changes were observed for PCB congener 153.

**Conclusions:**

These data suggest that PCB concentrations may change during the periconception interval, questioning the stability of persistent compounds during this critical window.

The environmental persistence and lipophilicity of polychlorinated biphenyls (PCBs) in the Great Lakes basin and elsewhere has lead to bioaccumulation within the aquatic food chain and human exposure (Ayotte et al. 1995; Bloom et al. 2005; He et al. 2001). Dietary PCB exposure has been associated with adverse reproductive (Buck et al. 2000; Mendola et al. 2005) and developmental outcomes (Jacobson and Jacobson 1997), underscoring the importance of exposures during critical or sensitive windows (Morford et al. 2004).

Although past research has focused on *in utero* PCB exposure for clinically recognized pregnancies in relation to human development, only limited investigation of periconception exposures has been undertaken (Chapin et al. 2004). This impairs our ability to accurately model the effects of pregnancy and/or lactation in assessing reproductive outcomes conditional on pregnancy. We assessed concentrations of PCB congeners from preconception through pregnancy and postpartum to better understand their dynamics over critical windows.

## Materials and Methods

### Study population and sample

We used a prospective cohort design to recruit women from the New York State Angler Cohort Study (NYSACS), a population-based cohort comprising licensed anglers 18–40 years of age who were randomly selected from 16 contiguous counties along Lakes Erie and Ontario (Vena et al. 1996). The purpose of this prospective pregnancy study with preconception enrollment was twofold: to obtain longitudinally collected biospecimens for the quantification of PCBs over sensitive critical windows, and to evaluate periconception data collection methodologies appropriate for population-based epidemiologic research. The study protocol complied with the U.S. regulations on the protection of human subjects; all study participants gave written informed consent before participation in any aspect of the study.

In 1996–1997, introductory recruitment letters were mailed to 2,637 female participants in the NYSACS who had stated interest in possibly becoming pregnant in 1995–1996. After repeated telephone attempts, 1,031 (39%) women were successfully screened, of whom 244 were eligible for participation—defined as planning pregnancy in the next 6 months, age 18–34 years, and no physician diagnosis of infertility. The study sample comprised 113 women (46%) who reported planning pregnancies within 6 months; however, 14 women were already pregnant and therefore excluded. The final study cohort comprised 99 women, of whom 20 withdrew over the course of the 12 months of attempting pregnancy. The distribution of reproductive outcomes among participating women completing the study included 54 (68%) women whose pregnancies resulted in live births, 10 (13%) women whose pregnancies ended in early losses, 4 (5%) women whose pregnancies ended after clinical recognition, and 11 (14%) women who were unable to conceive within 12 menstrual cycles of trying.

### Data collection

Participation required a baseline interview, completion of a daily diary, and provision of nonfasting blood specimens at baseline (preconception) and after a positive home pregnancy test result or after 12 unsuccessful menstrual cycles without pregnancy. For women giving birth, an additional blood specimen was obtained at approximately 6 weeks after delivery (postpartum). The research nurse instructed women in the proper use of home pregnancy kits reported to be capable of detecting 50 mIU of human chorionic gonadotropin (hCG).

Approximately 25 mL of blood yielding approximately 10 mL of serum were obtained as follows: from all 79 participating women at baseline or preconception; from 54 women after a positive pregnancy test resulting in a live birth (prenatal); from 10 women after a positive pregnancy test approximately 2 weeks postimplantation that resulted in an early pregnancy loss (EPL); from 4 women after a positive pregnancy test resulting in a clinical pregnancy loss (CPL); from 54 women approximately 6 weeks after a live delivery (postnatal); and from 10 women after 12 unsuccessful menstrual cycles without pregnancy (infertile). Thus, we have a blood specimen reflecting the varying critical windows (preconception, postimplantation, and postnatal) and reproductive outcomes (pregnancy loss, live birth, infertility). The blood samples were transported on ice to the toxicology laboratory; these included 73 (92%) baseline or preconception, 53 (98%) prenatal, 10 (100%) EPL, 3 (75%) CPL, 52 (96%) postnatal, and 9 (90%) infertility blood samples.

### Toxicologic analysis

Serum samples were mixed with solutions of International Union of Pure and Applied Chemistry PCB isomers no. 46 and 142 (surrogate standards) and left overnight to equilibrate (Greizerstein et al. 1997). Methanol was added to precipitate the proteins, and the resulting mixture was extracted with hexane in a rotating extraction device at 50 rpm for 20 hr. Samples were then centrifuged and the extract was concentrated under a slow stream of nitrogen at 50° C to 2 mL. The extract was placed on a deactivated Florisil column and eluted with hexane. The eluate was evaporated to a small volume under a slow stream on nitrogen using 200 μL isooctane as a keep solvent. Isomers no. 30 and 204 were added as internal standards to each extract. An aliquot of the mixture was injected into the gas chromatograph equipped with an electron capture detector (Agilent Technologies, Santa Clara, CA).

A quality control (QC) sample was made from a matrix blank consisting of sheep serum, which was spiked with 0.6 ng/g PCB congeners 6, 44, and 52; 0.3 ng congeners 101, 138, 153, 180, 185, and 205; and 0.3 ng pesticides dichlorodiphenyldichloroethylene, hexachlorobenzene, and mirex. The QC sample was run with each batch of 10 samples and appropriate reagent and matrix blanks. Quality control charts were kept throughout the study and any batch of samples in which the QC values exceeded acceptance criteria were rerun. Surrogate congener standards 46 and 142 were added to each sample to assess recoveries. The surrogate standard recoveries for one set of 500 serum samples was 85.5 ± 18.6% for PCB-46 and 83.1 ± 16.4% for PCB-142. Serum specimens were run in batches of 10 plus four quality control samples: reagent blank, matrix blank, matrix blank containing a mixed standard of 15 specific congeners and pesticide components at known values, and a duplicate participant sample. The laboratory is in compliance in the AMAP Ring Test Proficiency Program for Persistent Organic Pollutants in Human Serum (Centre de toxicologie Institut national de santé publique du Québec, Québec, Canada).

We corrected laboratory observed values only for recovery to minimize measurement error and potential biases associated with substitution patterns below the limits of detection (LOD) (Richardson and Ciampi 2003; Schisterman et al. 2006). *A priori,* 64 single-eluting and 12 di-eluting congeners were quantified and summed into three groupings (Cooke et al. 2001): *a*) total PCB congeners; *b*) estrogenic congeners (4 + 10, 8 + 5, 15 + 17, 18, 31, 44, 47, 48, 52, 70, 77 + 110, 99, 101, 126, 136, 153, and 188); and *c*) anti-estrogenic congeners (77 + 110, 105, 114, 118, 126, 156 + 171, and 169). We assessed PCB congener 153 individually for comparison with other work focusing on female fecundity (Axmon et al. 2001). We also assessed PCB-118 individually given that most concentrations were above the LOD, similar to PCB-153, and to aid in the interpretation of results by PCB grouping. We quantified total serum lipids (TL) using enzymatic methods as the function of total cholesterol (TC) and triglycerides (TG) expressed in milligrams per deciliter. We assumed free cholesterol to be 27% of the total, and predicted phospholipids from regression on total cholesterol where TL = 2.27 TC + TG + 0.623 (Phillips et al. 1989). For analysis purposes, PCB concentrations are reported as nanograms per gram wet weight with serum lipids entered as a covariate in the analytic model to minimize bias arising from automatic lipid adjustment of PCB concentrations (Schisterman et al. 2005).

### Statistical analysis

With descriptive statistics, many PCB congener distributions were not normally distributed even after Box-Cox transformations; therefore, no further transformations were undertaken. We used bivariate analysis to explore associations between the changes in concentrations from the baseline or preconception to the next serum measurement in relation to serum lipids (milligrams per deciliter) and relevant study covariates—i.e., body mass index (weight in kilograms/height in square meters), gravidity (number of pregnancies), parity (number of births), and lifetime duration of lactation in months (Glynn et al. 2003; Grimvall et al. 1997; Kostyniak et al. 1999; Sweeney et al. 2001).

We estimated Spearman rank correlation coefficients to describe correlations between baseline PCB groupings, and between the measurements across critical windows. For each correlation, we used the chi-square test to obtain the level of significance against the null (zero correlation), using Fisher’s *z*-transformation [*z* = 0.5 log(1 + *r*)/(1 – *r*)] with the appropriate variance correction.

We estimated the overall amount of change between measurements and the daily rate of change in serum PCB concentrations and total serum lipids for 48 paired preconception-prenatal specimens, 47 paired prenatal–postnatal specimens, 10 paired preconception–EPL specimens, and 9 paired preconception–infertility specimens, using the following algorithm:


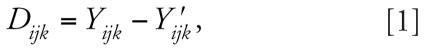


where *Y_ijk_* represents measures for the *i*th (1,……,*n*) participant; for the *j*th PCB grouping (where 1 = total PCBs, 2 = estrogenic PCBs, 3 = antiestrogenic PCBs, 4 = PCB-118, 5 = PCB-153, and 6 = total serum lipids variable, respectively); demonstrating the *k*th (1, 2, 3, 4) reproductive outcome (where 1 = prenatal or a pregnancy resulting in a live birth, 2 = EPL or a pregnancy resulting in an early loss, 3 = infertile or 12 menstrual cycles without conception, and 4 = postnatal after a live birth; and *Y*′*_ijk_* represents the baseline or preconception measurement for *k* = 1, 2, or 3, and the prenatal measurement from a pregnancy resulting in a live birth where *k* = 4.

Women’s daily rate of change, defined as the difference in the concentration between the first (baseline) and second specimen collection divided by the number of days between specimen collections, was derived as follows:


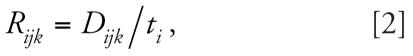


where *t_i_* represents the duration in days, for the *i*th (*1,…,n*) participant, between the reported positive hCG pregnancy test date and the outcome specimen sample date, where *k* = 1, 2, between the dates of the baseline and outcome specimens where *k* = 3, and the date of the prenatal and postnatal specimens where *k* = 4. We assumed no change in PCB concentration between the date of baseline measure and the date of the positive hCG test among those women who conceived. We further estimated the daily rates of change in concentrations from the time of the positive pregnancy test to the second blood specimen. Length of gestation at the time of the prenatal sample ranged from 8 to 126 days with a mean (± SD) of 31.4 ± 17.5 days and a median of 29 days.

We estimated the mean overall and daily rate of change in PCB concentration using multiple linear regression, adjusting for preconception PCB concentration and lipids, centering each by its mean to facilitate interpretation of model intercepts as the change in PCBs at the average lipid and baseline PCB concentrations. Estimating the difference in PCBs’ grouping concentrations between the two measurements simplified the variance structure by removing part of the correlation between observations on the same woman. Additional covariates were not included in models given the absence of significance, nor were separate models run for the three clinical pregnancy losses. Serum lipids could not be quantified for 10 women (8 live births, 1 pregnancy loss, and 1 infertile) given insufficient sample necessitating expectation maximization (Yuan 2000).

We evaluated the normality assumption for each regression model by examining residuals employing Q–Q plots and by comparing predicted values with those generated employing the LOESS nonparametric regression procedure, because changes in PCB concentrations deviated substantially from normality even with transformation. Significance was defined as *p* < 0.05 for two-tailed tests; crude differences in overall change and daily rate of change in PCB concentration were evaluated using the Wilcoxon signed rank test.

## Results

We observed no significant differences in the median preconception PCB concentrations by PCB grouping and reproductive outcome or for any of the other study covariates (Table 1). The median interval between the timing of the two serum PCB measurements was 63 days [interquartile range (IQR) = 34] for early pregnancy loss, 79 days (IQR = 76) for clinical pregnancy loss, 151 days (IQR = 180.5) for live birth, 446 days (IQR = 127) for infertility, and 240 days (IQR = 21) for the pre- and postnatal.

Baseline PCB groupings were significantly correlated with each other (Table 2). Significant correlations were found between baseline and prenatal measures for all PCB groupings except estrogenic PCBs, between baseline and early pregnancy loss measures for total PCBs, PCB-118 and PCB-153, and between pre- and postnatal samples for estrogenic PCBs and PCB-153 (Table 3).

Table 4 presents the adjusted regression models for overall and daily rate of change in concentrations by PCB grouping and reproductive outcome. *p*-Values are presented in lieu of 95% confidence intervals to avoid the use of negative exponents, though the latter are available on request. Significant mean overall and daily rates of change in PCB concentrations (nanograms per gram serum) were consistently observed for women becoming pregnant regardless of outcome for total, estrogenic, and antiestrogenic groupings. Results for the infertile women were inconsistent (positive and negative) and none were significant. Of particular note is the absence of any overall or daily changes in concentrations for PCB-153 regardless of reproductive outcome, in contrast to an observed significant change (–0.001) for daily PCB-118 among women giving birth. Overall mean PCB concentrations (nanograms per gram serum) adjusted for duration of breast-feeding (reported by 34 women) significantly increased in women from prenatal to postnatal measurement for total (1.938), estrogenic (0.628), and antiestrogenic PCBs (0.228) as did the daily rate of change—0.008, 0.003, and 0.001, respectively. Median serum lipids (expressed in milligrams per deciliter) declined 15.97 (range –282.89 to 261.29) from preconception to prenatal for women whose pregnancies resulted in a birth, and 1.57 (range –144.71 to 105.96) for women experiencing losses, but increased 18.97 (range –53.78 to 137.68) for infertile women. None of these results achieved significance (data not shown). Overall and daily rates of change in serum lipids were not significant predictors of changes in PCB concentrations (data not shown), except for the daily rate of change in anti-estrogenic PCBs between preconception and the prenatal blood and between the preconception and EPL measurements (i.e., β = 0.0004 and β = 0.0009, respectively; *p* = 0.02).

**Figure 1:**
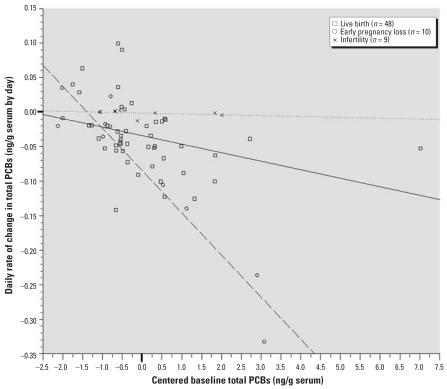
Daily rate of change in total PCB concentrations as a function of baseline PCB concentrations. Solid line, linear regression best fit line for live delivery group; dashed line, linear regression best fit line for early loss group; dotted line, linear regression best fit line for infertile group.

Figure 1 illustrates the daily rate of change in total PCBs by reproductive outcome as a function of women’s baseline concentrations. The dependence of daily rate of change on baseline concentration is illustrated underscoring the need to adjust for baseline concentration when estimating the mean daily rate of change. This finding supports the use of summary statistics beyond simple correlations to allow simultaneous modeling of important covariates.

## Discussion

This prospective pregnancy study with preconception enrollment of women is the first to demonstrate the possible instability of PCB concentrations over critical windows such as conception and implantation, as measured by hCG-detected pregnancy. Our findings suggest that both the overall and daily rate of change in serum PCB concentrations may be associated with reproductive outcome, though cautious interpretation is needed given the limited cohort size and reliance on only two measurements. Further supporting these observations is the lack of change in concentrations for women who failed to achieve an hCG-confirmed pregnancy within 12 menstrual cycles, though admittedly with a limited number of women. To this end, use of preconception cohorts with ascertainment of all women regardless of reproductive outcomes is essential for understanding exposure profiles relevant for the assessment of time-dependent health effects. Last, our study findings remained consistent regardless of PCB grouping suggesting that categorization did not drive our results. In the absence of a universally accepted classification scheme for the assessment of human health effects, our *a priori* classification allowed us to formalize our assumptions when characterizing the cohort’s exposure over time.

The reasons for the early changes between preconception and hCG-detected pregnancy are unknown and somewhat puzzling given the alleged persistence of these chemicals in the body. We speculate that early homeostatic changes after human conception and early embryonic development may affect chemical mobilization from lipid reserves, possibly as a result of the many physiologic changes accompanying pregnancy (Bernstein et al. 2001) that may affect the absorption, distribution, metabolism, and excretion of exogenous compounds (Casarett et al. 1996). Another possible interpretation is laboratory noise given the observed distribution of concentrations including values below the LOD [Appendix 1, Supplemental Material (online at http://www.ehponline.org/docs/2007/10086/suppl.pdf)]. We do not believe that this potential source of bias accounts for our findings, given our use of observed values as previously discussed.

Regardless of the underlying mechanisms, these findings underscore the potential for variability arising from epidemiologic studies that rely on a single blood measurement of exposure, especially if captured at varying times during pregnancy or if relying only on women with clinically viable pregnancies. The utility and feasibility of prospective pregnancy studies with preconception enrollment of women or couples has been discussed previously (Buck et al. 2004). In essence, pregnancy loss may be viewed as a competing risk for birth if higher exposures or exposures during critical windows systematically are associated with pregnancy outcome. Bias also may arise from collider stratification (Hernan et al. 2004) that may arise when an intermediate variable such as reproductive outcome is used to stratify the analysis. Because reproductive outcome could be directly affected by two or more other unmeasured variables, stratifying on it can generate spurious study findings. However, it is unlikely that this type of bias would explain the large differences observed in our study. Still, we recognize the need for the results to be interpreted within the context of this validity threat. Our results discourage continued reliance on a single PCB congener such as PCB-153 for assessing the reproductive or developmental toxicity of all PCBs or other persistent compounds in the absence of further empirical assessment.

We are unaware of any prospective pregnancy studies with preconception enrollment that quantified serum PCBs concentrations across critical windows. To this end, we believe our longitudinal cohort study is the first to provide empirical data regarding mean changes in serum concentrations of PCBs from the preconception-to-postnatal window of human development. A few previous investigators have assessed the stability of select PCB congeners across trimesters of clinically established pregnancies and reported them to be highly correlated (Longnecker et al. 1999), as do investigators comparing maternal concentrations during established clinical pregnancies with cord or postnatal concentrations (Ayotte et al. 2003; Jarrell et al. 2005). However, such correlation coefficients cannot be adjusted for time intervals or other relevant covariates. The median prenatal total PCB concentration for the 48 women giving birth in our study is comparable to that for the 67 first trimester women in the study by Longnecker et al. (1999), suggesting similarity in study cohorts with regard to exposure status (4.5 ng/g serum and 4.4 μg/L serum, assuming nanograms per gram ≈ micrograms per liter ≈ parts per billion).

Of added note is the capture of all hCG pregnancies in our study with the use of home pregnancy test kits and women failing to conceive along with clinical pregnancies. *A priori,* we were interested in obtaining both pre- and postconception biospecimens with an additional biospecimen after an untoward reproductive outcome or birth for fertile women. Thus, we had only one blood sample obtained during pregnancy. We also recognize that some pregnancies may have been undetected by home pregnancy tests, possibly resulting in women being misclassified as infertile, despite the absence of significant differences in baseline concentrations by reproductive outcome. Not all women’s blood specimens could be precisely collected on the same day, given that women resided in 16 counties in New York State with a single research nurse available for home blood collection. Timing of the second sample also depended on the woman’s compliance with home pregnancy testing and her ability to accurately recognize and report a positive test. Given the educational attainment of the women coupled with instruction by the nurse with regard to fertility awareness and the proper use of home pregnancy test results, pregnancy recognition bias is not likely to have affected the timing of the test. Our analyses do consider the interval (in days) between our measurements. We decided *a priori* not to correct for multiple comparisons given the exploratory nature of this work and our intent to try to globally assess patterns within and between PCB groupings and by reproductive outcomes. Finally, we remain uncertain about how best to model the chemical mixtures more representative of women’s actual exposures. Our initial attempt to categorize PCB congeners in an *a priori* manner should be viewed as preliminary and needing further refinement as our understanding of the biological activity and underlying mechanisms evolves for this class of compounds.

## Conclusions

In sum, PCB concentrations declined significantly during the periconception window of human development among women achieving pregnancy. PCB concentrations increased among women with postnatal measurement. These findings suggest a relatively dynamic nature of serum PCB concentrations during the earliest windows of human development, underscoring the need to characterize exposures during the periconception window.

## Figures and Tables

**Table 1:** Description of study cohort at baseline by reproductive outcome [median (range)].

Characteristic	Prenatal (*n* = 48)	Pregnancy loss (*n* = 10)	Infertility (*n* = 9)
PCBs (ng/g serum)			
Total PCBs	5.27 (3.66–12.68)	5.21 (3.89–9.09)	4.62 (4.10–7.19)
Estrogenic PCBs	2.29 (1.66–3.77)	2.29 (1.78–4.54)	2.09 (1.79–3.18)
Antiestrogenic PCBs	0.20 (0.03–0.65)	0.18 (0.03–0.33)	0.22 (0.12–0.29)
PCB-118	0.09 (0.01–0.31)	0.12 (0.02–0.16)	0.08 (0.05–0.21)
PCB-153	0.26 (0.10–1.24)	0.25 (0.16–0.47)	0.24 (0.15–0.55)
Total serum lipids (mg/dL)	530.17 (357.57–915.38)	529.05 (307.91–829.94)	475.39 (414.45–723.67)
Age (years)	30.5 (26–34)	29.5 (26–33)	30.0 (26–32)
BMI (kg/m^2^)	22.18 (17.7–39.9)	22.39 (17.54–34.56)	21.46 (17.28–31.15)
Gravidity (no. of pregnancies)	1 (0–5)	1 (0–3)	0.5 (0–2)
Parity (no. of births)	1 (0–3)	1 (0–3)	0 (0–2)
Breast-feeding (no. of months)^a^	6.0 (1–28)	10.5 (2–13)	13.0 (6–20)

BMI, body mass index. None of the above differences achieved statistical significance. Reproductive outcome refers to the timing of the second blood collection.

a Restricted to the 39 women reporting a history of breast-feeding at baseline.

**Table 2:** Spearman correlation coefficients among baseline PCB groupings (*n* = 67).

PCB grouping	Total PCBs	Estrogenic PCBs	Antiestrogenic PCBs	PCB-118	PCB 153
Total	1.000	0.938**	0.494**	0.341*	0.600**
Estrogenic		1.000	0.343*	0.258*	0.499**
Antiestrogenic			1.000	0.799**	0.629**
PCB-118				1.000	0.418*
PCB-153					1.000

* *p* < 0.05

** *p* < 0.0001.

**Table 3:** Spearman correlation coefficients among paired biospecimens by PCB grouping and critical window.

PCB grouping	Baseline measure with prenatal (*n* = 48)	Baseline measure with early loss (*n* = 10)	Baseline measure with infertility (*n* = 9)	Prenatal measure with postnatal (*n* = 47)
Total PCBs	0.328*	0.745*	0.317	−0.265
Estrogenic PCBs	0.168	0.588	0.383	−0.346*
Antiestrogenic PCBs	0.558**	0.394	0.467	0.095
PCB-118	0.755**	0.770*	0.300	0.281
PCB-153	0.513*	0.770*	0.567	0.320*

* *p* < 0.05

** *p* < 0.0001.

**Table 4:** Mean adjusted overall and daily rates of change in serum PCB concentrations, by PCB groupings and reproductive outcome.

	PCB grouping	Prenatal^a^ (*n* = 48)	Pregnancy loss^a^ (*n* = 10)	Infertility^a^ (*n* = 9)	Prenatal to postnatal^b^ (*n* = 47)
Overall change (ng/g serum)	Total PCBs	−1.012**	−1.452**	−0.281	1.938*
	Estrogenic PCBs	−0.444**	−0.647**	−0.312	0.628*
	Antiestrogenic PCBs	−0.106**	−0.093*	0.065	0.228*
	PCB-118	−0.016	−0.014	0.010	0.023
	PCB-153	−0.021	−0.041	−0.015	0.016
Daily rate of change (ng/g serum)	Total PCBs	−0.034**	−0.085**	−0.000	0.008*
	Estrogenic PCBs	−0.016**	−0.040**	−0.001	0.003*
	Antiestrogenic PCBs	−0.004**	−0.004#	0.000	0.001*
	PCB-118	−0.001*	−0.001	0.000	0.000##
	PCB-153	−0.000	−0.003	−0.000	0.000##

a Adjusted for overall change (mg/dL serum) or daily rate of change (mg/dL serum) in total serum lipids and baseline PCB concentration (ng/g serum).

b Adjusted for overall change or daily rate of change in total serum lipids (mg/dL), prenatal PCB concentration and duration of breast-feeding between delivery and the postnatal sample.

* *p* ≤ 0.004

** *p* < 0.0001;

# *p* = 0.03.

## Observed values were 0.000115 and 0.000053, respectively, but rounded to zero.
